# Folie a deux / induced delusional disorder – case report and literature review

**DOI:** 10.1192/j.eurpsy.2023.2202

**Published:** 2023-07-19

**Authors:** A. Lourenço, A. L. Falcão, G. Soares, J. Petta, C. Rodrigues, M. Nascimento, C. Oliveira

**Affiliations:** Clínica 3, Centro Hospitalar Psiquiátrico de Lisboa, Lisboa, Portugal

## Abstract

**Introduction:**

*Folie a deux*, also known as shared psychotic disorder or induced delusional disorder, is a rare mental disorder that was first described in France in the late 19^th^ century and was referred to delusions shared between two individuals in close relationship. The concept has evolved and according to ICD-10 the following criteria for the diagnosis is phenomenology-based only.

**Objectives:**

To describe a clinical case and review the existing evidence on *folie a deux.*

**Methods:**

Clinical case and non-systematic review of the literature, from the last 15 years, on folie a deux. For this research, the keywords “*folie a deux*”, “shared psychotic disorder” and “induced delusional disorder” were used in the MEDLINE/PubMed database.

**Results:**

The clinical case presented refers to a 56-year-old female patient with no known psychiatric history. The patient stated that 5 years ago when his mother died, neighbors began to persecute her and her sister. She was medicated with a second-generation antipsychotic without total remission of symptoms. Generally, in *folie a deux* there is a close and prolonged relationship between the inducer and the receptor, as described in this case. We considered that the sister is the active subject. The delusion is persecutory, the most common in this disorder. The patient kept her job until she was hospitalized and as described in the literature patients with folie a deux maintain their functionality, which is responsible for the underdiagnosis of this disorder. The fact that the current evidence is based on case reports reflects the underdiagnosis and rarity of this disorder.

**Conclusions:**

This clinical case highlights the challenging diagnosis and difficulty in treating this condition. Patients can be diagnosed many years after the onset of symptoms, which may not resolve with treatment. Much information, as prevalence, natural history, and optimal treatment, is lacking on folie a deux, and the etiology remains unknown. As such, prospective studies should be carried out to help understand this disorder.

**Disclosure of Interest:**

None Declared

